# Distinct expression patterns of the E3 ligase SIAH-1 and its partner Kid/KIF22 in normal tissues and in the breast tumoral processes

**DOI:** 10.1186/1756-9966-29-10

**Published:** 2010-02-09

**Authors:** Heriberto Bruzzoni-Giovanelli, Plinio Fernandez, Lucía Veiga, Marie-Pierre Podgorniak, Darren J Powell, Marco M Candeias, Samia Mourah, Fabien Calvo, Mónica Marín

**Affiliations:** 1 Laboratoire de Pharmacologie Expérimentale et Clinique, Université Paris 7 Denis Diderot, Département Cibles Pharmacologiques dans les Cancers, INSERM U940 (ex U716), 27, rue Juliette Dodu, 75010 Paris, France; 2 Centre d'Investigations Cliniques INSERM 9504 - AP-HP. Hôpital Saint-Louis, 1, Av. Claude Vellefaux, 75010 Paris, France; 3 Sección Bioquímica, Facultad de Ciencias, Iguá 4225, Montevideo, Uruguay; 4 Current address: Department of Clinical Biochemistry, Salford-Royal Hope Hospital, Manchester, UK

## Abstract

SIAH proteins are the human members of an highly conserved family of E3 ubiquitin ligases. Several data suggest that SIAH proteins may have a role in tumor suppression and apoptosis. Previously, we reported that SIAH-1 induces the degradation of Kid (KIF22), a chromokinesin protein implicated in the normal progression of mitosis and meiosis, by the ubiquitin proteasome pathway. In human breast cancer cells stably transfected with SIAH-1, Kid/KIF22 protein level was markedly reduced whereas, the Kid/KIF22 mRNA level was increased. This interaction has been further elucidated through analyzing SIAH and Kid/KIF22 expression in both paired normal and tumor tissues and cell lines. It was observed that SIAH-1 protein is widely expressed in different normal tissues, and in cells lines but showing some differences in western blotting profiles. Immunofluorescence microscopy shows that the intracellular distribution of SIAH-1 and Kid/KIF22 appears to be modified in human tumor tissues compared to normal controls. When mRNA expression of SIAH-1 and Kid/KIF22 was analyzed by real-time PCR in normal and cancer breast tissues from the same patient, a large variation in the number of mRNA copies was detected between the different samples. In most cases, SIAH-1 mRNA is decreased in tumor tissues compared to their normal counterparts. Interestingly, in all breast tumor tissues analyzed, variations in the Kid/KIF22 mRNA levels mirrored those seen with SIAH-1 mRNAs. This concerted variation of SIAH-1 and Kid/KIF22 messengers suggests the existence of an additional level of control than the previously described protein-protein interaction and protein stability regulation. Our observations also underline the need to re-evaluate the results of gene expression obtained by qRT-PCR and relate it to the protein expression and cellular localization when matched normal and tumoral tissues are analyzed.

## Introduction

SIAH-1 and SIAH-2 are human homologues of the *Drosophila seven in absentia *(sina) gene [1]. E3 ligase activity is the best characterized function of the family of SIAHs proteins [2,3]. SIAH proteins contain an N-terminal RING domain that binds E2 proteins and a C-terminal substrate binding domain that interacts with their target proteins, tagging them with Ubiquitin, thereby targetting their degradation by the ubiquitin-proteasome pathway [2-4]. The human SIAH-1 protein is 282 amino acids long, and was found to oligomerize via its C-terminal sequences [5,2]. The protein structure also contains two zinc finger cytokine-rich domains and shares 77% identity with SIAH-2 [5].

Numerous substrates targeted for degradation by SIAH proteins have been reported; examples include netrin-1 receptor/deleted in colorectal cancer (DCC) [6], the nuclear receptor co-repressor (N-CoR) [7], the transcriptional activator BOB.1/OBF.1 [8,9], c-Myb [10], Kid [3] and CtIP [11]. RING finger proteins have also been shown to regulate their own stability through proteasomal degradation [2].

Interestingly, not all SIAH-binding proteins are targets of SIAH-mediated degradation, as it occurs for α-tubulin [3], Vav [12], BAG1 [13] and others proteins [14]. SIAH-1 is also implicated in GAPDH transport to the nucleus in a novel cell death cascade, suggesting that SIAH proteins may play additional roles in cell biology [15].

It has been shown that the mRNAs of these two proteins are widely expressed but at different levels in several normal and neoplasic human tissues [5,16]. SIAH-1 mRNA was found highly expressed in placenta, skeletal muscle and testis and also in some cell lines, however, there is a paucity of data concerning endogenous SIAH-1 protein expression in human cells and tissues [17].

Our previous observations led us to propose that SIAH-1 could have a role in tumor suppression and apoptosis [5,17,18]. In fact, the murine SIAH-1 was identified as a p53 inducible gene, which is up-regulated during the physiological program of cell death [19]. The human SIAH-1 is activated during tumor suppression and apoptosis, notably during physiological apoptosis occurring in the intestinal epithelium [17]. We also reported that over-expression of SIAH-1 in the epithelial breast cancer cell line MCF-7 blocked cellular growth by altering the mitotic process, predominantly during nuclei separation and cytokinesis, leading to multinucleated giant cell formation and tubulin spindle disorganization [17].

In order to elucidate the role of SIAH-1 in the cell and the mechanisms by which SIAH-1 interferes with the mitotic process, we previously searched for SIAH-1-interacting proteins using the yeast two-hybrid system [3]. Amongst other proteins, we identified Kid (KIF22), a chromosome and microtubule binding-protein implicated in chromosomal positioning and segregation during cell division [20,21]. We showed a clear regulatory link between both proteins since SIAH-1 was involved in the degradation of Kid/KIF22 via the ubiquitin proteasome pathway [3]. Further evidence implicating SIAH-1 in tumor suppression was shown to be related to its role in the regulation of β-catenin [22] and hypoxia-inducible factor 1α (Hif-1α) [23,24].

Despite these efforts, the role of SIAH-1 as a tumor suppressor remains controversial since many efforts to identify putative mutations associated with tumoral processes have been almost unsuccessful. Medhioub *et al*. [25] searched for somatic mutations in different human tumors and Matsuo *et al*. [26] analyzed human hepatocellular carcinomas (HCCs); both authors failed to detect any somatic mutations in SIAH-1. In recent works, Kim *et al*. [27] found two missense mutations in the SIAH-1 gene in gastric cancer and Brauckhoff *et al*. [28] observed a reduced expression of SIAH-1 in HCCs.

Therefore, these few studies undertaken to establish a correlation between changes either in the sequence or expression of SIAH-1 with tumoral processes have been inconclusive. This study has attempted to further our understanding by analyzing mRNA and protein expression of SIAH-1 and it's substrate Kid/KIF22, in both normal and tumor tissues. The results suggest that regulation of the stability for these two interacting proteins could be complemented by an additional regulation of their mRNA levels.

## Materials and methods

### Patients and tissue samples

Breast cancer tissues and corresponding non-cancerous breast tissues were obtained after informed consent from patients who underwent breast resection in hospitals from Montevideo (Uruguay), between 2000 and 2004. The study included 50 post menopause females aged between 42 and 90 years with a median age of 66 years. The samples were anonymized before the study. A pathologist dissected tissue samples from surgical specimens and confirm quality of tissues microscopically. Cancerous and non-cancerous tissues were immediately frozen and stored at -80°C until analysis.

### Antibodies

Polyclonal antibodies against an N-terminal peptide of SIAH-1 produced in chicken were used as previously described [17]. Polyclonal antibodies against Kid/KIF22 were produced in rabbit with purified GST-Kid/KIF22 protein (Agrobio, France).

### RNA extraction and cDNA synthesis

Total RNA from frozen tissues were extracted with Trizol Reagent (Invitrogen) following manufacturer's instructions. The quality of RNA was analyzed by electrophoresis on agarose gels stained with ethidium bromide. One microgram of total RNA was reverse-transcribed in a final volume of 20 μL containing 1 × reverse transcriptase buffer (Invitrogen), 1.25 mM dNTPs (Quantum Biotechnologies) 10 mM DTT (Invitrogen), 5 ng/μL random hexamers (Roche), 1 U/μL RNAse inhibitor and 10 U/μL M-MLV reverse transcriptase (Invitrogen). The reaction mix was incubated at 42°C for 1 h, the reverse transcriptase was inactivated by 5 min incubation at 95°C. cDNA was stored at -20°C until analysis.

### Quantitative Real-Time PCR

To evaluate the relative expression of SIAH-1 and Kid/KIF22, quantitative real time PCR was performed using a LightCycler (Roche Diagnostics). Primers and fluorescent TaqMan probes were designed using Primer Express software (PE Applied Biosystems) and are shown in Table 1. RT-PCR reaction were carried out with an aliquot of 2 μL of the resulting cDNA in a final volume of 20 μL, using 100 nM of the specific hydrolyzed probe, 200 nM of the probe flanking appropriate primer pairs, and 18 μL of LC fast start DNA master mix (Roche). After 10 min at 95°C, 45 cycles of 5 s at 95°C and 10 s at 60°C were performed. Standards were prepared from total normal RNA, amplified by RT-PCR and quantified. The concentrations of unknown samples were then calculated by setting their crossing points to the standard curve. The expression levels of SIAHs and Kid/KIF22 were normalized using the expression of the housekeeping gene *β*2 microglobulin as a reference. All experiments were performed in duplicate. All coefficients of variation of Cp values were < 1%.

**Table 1:** Primers and TaqMan probes used to quantify mRNA expression of SIAH-1, Kid/KIF22 and *β*2 microglobulin.

Gen	PCR Product (bp)	Primers	TaqMan Probe
SIAH-1	75	5'-GCTAAATGGTCATAGGCGACG-3' 5'-ATGGCTGTTGCAATTCCTTCAT-3'	5'-TTGACTTGGGAAGCGACTCCTCGATCTA-3'

Kid/KIF22	76	5'-CGGCCTTTTACCAATGAGAGC-3' 5'-GACCAAGCAATTCTTTCTGAGACA-3'	5'-CAGCCTCATGCCTTGGGACCTGTTAAG-3'

*β*2 microglobulin	67	5'-CGCTCCGTGGCCTTAGC-3' 5'-GAGTACGCTGGATAGCCTCCA-3'	5'-TGCTCGCGCTACTCTCTCTTTCTG-3'

### Western blotting

Cell lysates from 14 human cell lines and 10 normal human tissues were obtained from Imgenex (Clinisciences, France). 10 μg of protein lysates were resolved by reducing 12% SDS-PAGE and transferred to nitrocellulose membranes Hybond-C (Amersham). After electrophoresis, protein transfer was verified by Ponceau staining. The nitrocellulose membranes were probed with antibodies anti-SIAH-1 and anti-Kid/KIF22 (both diluted 1:1000) followed by horseradish peroxidase-coupled secondary antibodies (Jackson ImmunoResearch Laboratories, Inc.) anti-chicken IgG (diluted 1:2000) or anti-rabbit IgG (diluted 1:2500) and detected using a chemiluminescence-based detection system (ECL, Amersham).

### Immunofluorescence staining

Paraffined tissue array slides containing 20 normal and 19 matched malignant human tumor tissues, or 25 cancerous and 4 normal breast human tissues were obtained from Imgenex (Clinisciences, France), and processed as per manufacturer recommendations. Breast tumors and normal surrounding tissues from the same patients were obtained by sectioning frozen tissues. The slides were fixed in 2% paraformaldehyde (PFA) for 10 min at room temperature (RT) and washed in PBS six times. Nonspecific protein binding was blocked by incubation in a PBS solution containing 3% BSA, 0.1% saponin for 2 h at RT. Slides were then incubated overnight with primary antibody diluted in 0.3% BSA, 0.1% saponin in PBS at 4°C. After six washes with PBS, staining was revealed using a Rhodamine Red-X-conjugated secondary antibody for SIAH-1 and FITC-conjugated secondary antibody for Kid/KIF22 (Jackson Labs). Slides were subsequently analysed using a Zeiss epifluorescence microscope equipped with a cooled three-charged coupled device (3CCD) camera (Lhesa, France), triple band pass filter and a high numerical aperture lens (40 × 1.3 NA and 100 × 1.3 NA).

## Results

### Analysis of SIAH-1 in human tissues and cell lines extracts

**Figure 1:**
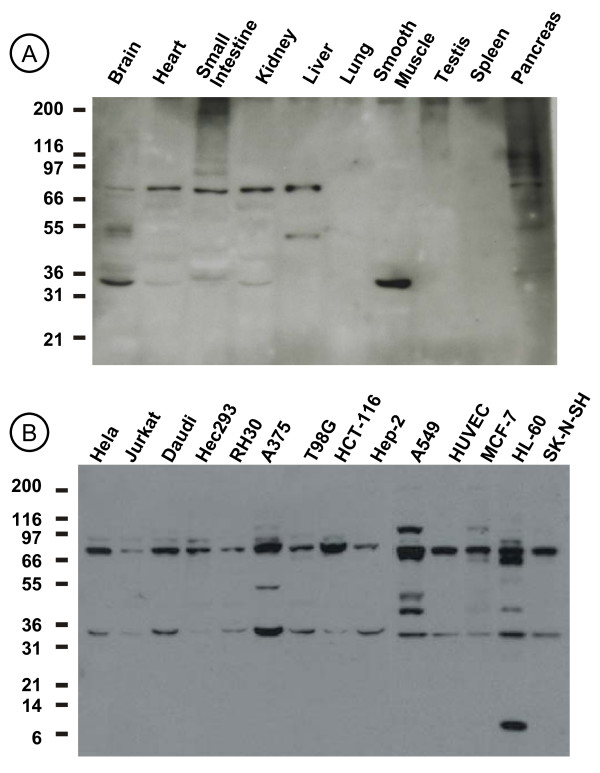
**Physiological expression of SIAH-1 protein**. Polyclonal chicken anti-SIAH-1 antibodies was used to detect SIAH-1 protein. (**a**) Immunoblot of protein extracts from different human tissues. (**b**) Immunoblot of different human cell lines derived from cervical carcinoma (HeLa), T-cell leukemia (Jurkat), Burkitt's lymphoma (Daudi), embryonal Kidney (293), rhabdomyoscarcoma (Rh30), melanoma (A375), glioblastoma (T98G), colon carcinoma (HCT-116), larynx carcinoma (Hep-2), lung carcinoma (A549), endothelial normal cells (HUVEC), breast adenocarcinoma (MCF-7), promyelocytic leukemia (HL-60) and bone marrow neuroblastoma (SK-N-SH).

The expression of SIAH-1 in a variety of human tissues and human derived cell lines was explored by western blotting using SIAH-1 anti-sera (previously described [17]), (Figure 1). Two major bands with an apparent MW of ~35 kDa and ~70 kDa were detected in human brain, heart, small intestine, Kidney and pancreas extracts. In contrast, no bands were evident in human lung, testis and spleen extracts (Figure 1a). The smooth muscle extract showed only the minor band of ~35 kDa. In addition, an extra band of ~52 kDa was detected in brain, liver and pancreas extracts. Besides the two principal bands, additional bands of higher molecular weight showing a ladder pattern were detected in small intestine and pancreas extracts. This profile is characteristic of polyubiquitinated proteins. In the cell lines homogenates we also found two main bands corresponding to the ~35 kDa and the ~70 kDa polypeptides (Figure 1b). In addition, other bands were detected in some cell lines like A375, A549 and HL60.

### SIAH-1 and Kid/KIF22 protein expression in cancerous and non-cancerous tissues

**Figure 2:**
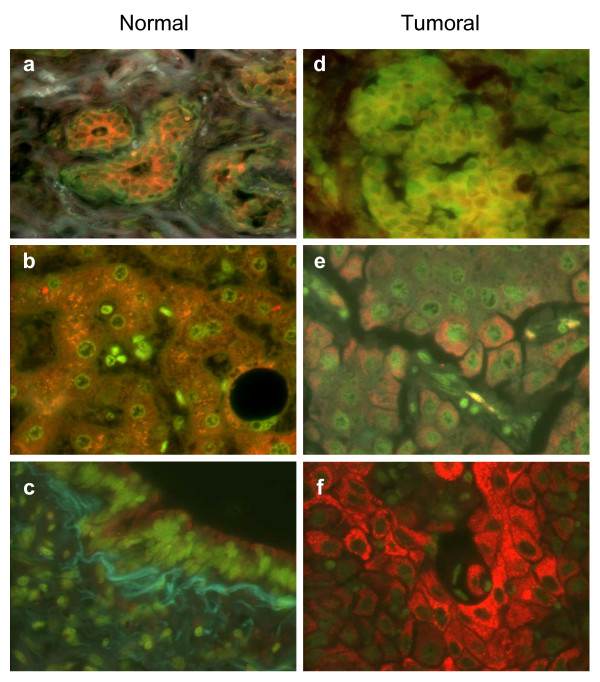
**SIAH-1 protein expression in normal and tumor tissues**. Normal breast (**a**), liver (**b**) and lung (**c**) normal tissues and its respective tumor counterpart from the same patient (**d**), (**e**) and (**f**) are showed. Paraffined tissues were stained with anti-SIAH-1 antibody, and detected with a secondary antibody conjugated to Rhodamine Red-X. Cells were counterstained with DioC6 (green) to mark the ER, cellular membranes, and mitochondria.

In order to further characterize SIAH-1 and Kid/KIF22 expression in cancerous and non-cancerous tissues, proteins were analyzed at the cellular level, by fluorescence microscopy. Firstly SIAH-1 staining on tissue array slides containing normal and matched malignant human tissues was performed. Comparing SIAH-1 expression in these tissues, it was shown that in the normal cells of most tissues the protein was predominantly expressed in the cytoplasm, showing a punctuate staining pattern. In normal breast tissues, acinar cells show a very strong label compared to surrounding cells (Figures 2a). In breast tumor tissues SIAH-1 expression was less intense and more heterogeneous showing a more diffuse pattern, and nuclei were also frequently stained (Figure 2d). In normal liver cells, SIAH-1 expression was also high and the expression was similar in all cells (Figure 2b). However, liver tumor tissues showed significant heterogeneity in SIAH-1 protein expression with some cells expressing high levels whereas in the majority there was no detectable expression (Figure 2e). Other analyzed organs displayed a less systematic variation between normal and tumor tissues (e.g. lung), however all the tumoural specimens displayed the heterogenous pattern, with groups of tumor cells expressing very high levels of SIAH-1 and others without any detectable expression (Figure 2f). In addition, very low levels of SIAH-1 protein were detected in some normal tissues (e.g. lung, Figure 2c) and is consistent with the Western blot findings in Figure 1a.

**Figure 3:**
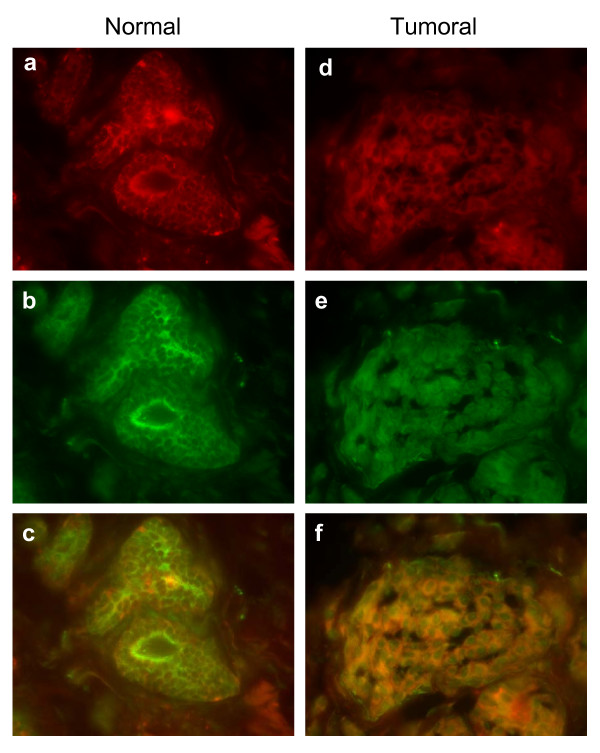
**Expression of SIAH-1 and Kid/KIF22 in paired normal and tumor breast tissues from the same patient**. Normal (**a**, **b **and **c**) and tumor (**d**, **e **and **f**) frozen breast tissue from the same patient are showed. (**a) **and (**d) **show SIAH-1 expression (detected as in Figure2), (**b) **and (**e) **show Kid/KIF22 expression detected using polyclonal chicken anti-Kid/KIF22 and anti-chicken-FITC as secondary antibody. (**c) **and (**f) **are overlay images of its respective SIAH-1 and Kid/KIF22 expression.

SIAH-1 and Kid/KIF22 protein expression were compared in normal and tumor breast tissues obtained from the same patient (Figure 3). The staining with SIAH-1 in normal tissues revealed a similar punctuate pattern as described above, while in tumor tissues the expression was diffuse and was both cytoplasmic and nuclear (Figure 3a, d). In the case of Kid/KIF22, the cellular labeling was also different between each normal tissue sample and its tumor counterpart (Figure 3b, e). In normal cells, the protein was mostly cytoplasmic, localized in perinuclear areas (Figure 3b), while in malignant cells the expression was more diffuse (nuclear and cytoplasmic), with a punctuate pattern observed mostly in nuclei (Figure 3e).

### SIAH-1 and Kid/KIF22 mRNA expression in normal and tumor tissues

We have previously analyzed the effect of SIAH-1 on Kid/KIF22 protein expression in MCF-7 cells stably transfected with SIAH-1 cDNA. The level of endogenous Kid/KIF22 protein was markedly reduced in clones overexpressing SIAH-1, whereas by Northern blot analysis we did not observe a reduction in Kid/KIF22 mRNA synthesis but rather an increase [3].

**Figure 4:**
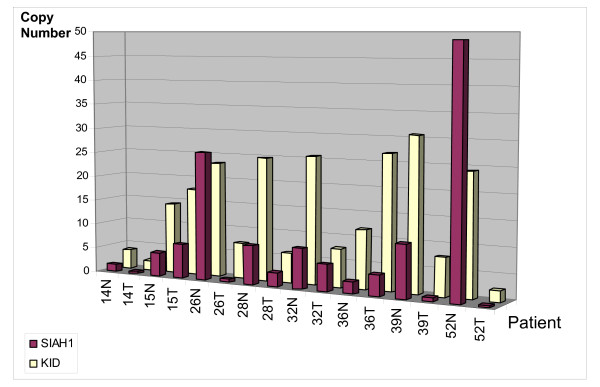
**SIAH-1 and Kid/KIF22 mRNA expression in paired normal and tumor breast tissues from the same patients**. Normal (N) and tumor (T) tissues from 8 patients representative of the study group are shown with their corresponding SIAH-1 (purple) and Kid/KIF22 (yellow) mRNA expression levels analyzed by rtPCR.

To further the relationship of Kid/KIF22 and SIAH-1 mRNA expression in physiological conditions and in tumoral processes, a quantitative RT-PCR of SIAH-1 and Kid/KIF22, in paired normal and cancerous breast tissues from the same patient was ran. Overall, samples were obtained from 50 patients, however mRNA quantification of coupled samples was only possible for 25 due to the low yield or poor quality of the extracted RNA from some of the tissues. The mRNAs were normalized related to the number of mRNA copies of the housekeeping gene β2 microglobulin. Important variations in the number of mRNAs copies amongst the samples were observed. Representative results from some of studied patients are showed in Figure 4. The number of SIAH1 copies extends from 1,48 to 61,6 × 10^3 ^(with a median of 17,41 × 10^3^) for normal tissues and from 0.35 × 10^3 ^to 52,04 × 10^3 ^copies (with a median of 5,73 × 10^3^) in tumoral tissues. Comparison of the paired normal and tumoral samples from patients, revealed that in 19 of 25 cases (76%), the level of SIAH-1 mRNA was reduced in breast cancer tissues compared to their corresponding non-cancerous breast tissue. In some of the samples the mRNA expression was remarkably reduced, more than 90% and in most cases the decrease was higher than 50%.

Interestingly, the changes in the levels of Kid/KIF22 mRNA mirrored that of SIAH-1 in all of the patients. Kid/KIF22 mRNA levels were decreased in all tumors in which SIAH-1 mRNA was decreased and vice versa (Figure 4). Moreover, except for one sample, the number of Kid/KIF22 mRNA copies was consistently higher than the SIAH-1 mRNA copies in all normal tissues (with a median of 19,2 × 10^3^) compared to their corresponding paired tumor tissues (median of 16,5 × 10^3^).

## Discussion

In this study, we compared SIAH-1 mRNA and protein expression levels in normal and tumor tissues and cell lines. SIAH-1 protein was found to be widely expressed in human cell lines and tissues. In non-proliferating tissues that express higher levels of SIAH-1 mRNA, a single band of the expected MW is detectable (muscle), or it represents almost the totality of the detected protein (brain). In other tissues and majority of cells lines a second band appears whose molecular weight is approximately the double of the first one. Although it is known that SIAH-1 forms stable homodimers [2,3,29], under reducing conditions used in SDS-PAGE a single band would be expected. The additional bands observed in Figure 1 could correspond to post-translational modifications, or to transcriptional or splicing variants of SIAH-1. Indeed, human SIAH-1 mRNA is 2.3 kb but an additional transcript of 2.5 kb was shown in placenta [5]; in MCF-7 cells, a SIAH-1 variant that encodes a 298 amino acid protein designated SIAH-1L was reported [30] whereas another variant named SIAH-1S encoding a 195 amino acid protein was detected in breast, Kidney and esophagus cancer tissues [31].

The broad tissue distribution of SIAH-1 suggests that it may play a relevant cellular role; however, high levels and splicing variants of SIAH-1 in particular tissues may represent sites of critical gene function or relate to physiological/pathological situations. Consistent with this, important differences in SIAH-1 expression were observed amongst cell lines and tissues. Interestingly, in some tissues such as the small intestine, other bands of high molecular weight appear suggesting the presence of polyubiquitinated forms of SIAH-1. This observation is consistent with previous reports, since SIAH-1 was shown to be auto-ubiquitinated and degraded via the proteasome pathway [2,3] and we showed a strong SIAH-1 expression in the cells at the apical of the intestine villi, where cells are differentiated and die by apoptosis [17].

By fluorescence microscopy, SIAH-1 was shown to be highly expressed in the cytoplasm of normal breast cells, with a punctuate pattern. In tumor tissues however, it appeared as a more uniform distribution, localized to both the cytoplasm and nucleus. Similarly, whereas in normal liver the expression was high and homogeneous among cells, tumor tissues showed significant heterogeneity with some cells expressing high levels of SIAH whilst being undetectable in others. Our results are consistent with previous reports on the cellular localization of SIAH-1; The punctuate pattern was described for endogenous SIAH-1 in human intestine and MCF-7 cell line [17] and GFP-SIAH-1 transfected cells [11], and was associated with the presence of this protein on early endosomes in PC12 cells [32]. Some authors have observed that in transfected cell lines overexpressing SIAH-1, the protein was localized predominantly in the cytoplasm [6,16,33], whilst others reported that it was also present in the nucleus [13] and particularly associated to the nuclear matrix [17]. It is interesting to note that regardless if SIAH-1 was expressed predominantly in cytoplasm or in the nucleus it showed the same punctuate pattern as we observed in our results. Other data showed that SIAH-1 was highly expressed in the nucleus, and that transient expression of cytoplasmic SIAH-1 resulted in a marked increase in apoptotic cells in hepatocellular carcinoma cell lines [26,28]. In addition, inhibition of nuclear SIAH-1 expression resulted in reduced tumor viability and deregulation of several genes involved in cell cycle regulation. These observations suggested a dual role for SIAH-1 in hepatocarcinogenesis depending on its expression level and subcellular localization. High-level expression in the cytoplasm could be related to tumor cell apoptosis, whilst reduced expression and nuclear accumulation correlates with tumor cell proliferation [26,28]. When other tissues were analyzed we observed a less systematic variation between normal and tumor tissues For example in normal lung tissue samples only very low levels of SIAH-1 were detected, in contrast to the paired tumoral counterparts which displayed a heterogeneous pattern with some cells expressing very high levels of SIAH-1. These data underline the need to correlate results obtained from tissues extracts with individual cell expression patterns viewed by immunochemistry.

SIAH-1 has also been implicated in the cytoplasm-nuclear translocation of Glyceraldehyde-3-phosphate dehydrogenase (GAPDH), a classic glycolytic enzyme and multi-functional protein [15]. GAPDH participates in a recently described cell death cascade in which a variety of stimuli activate the nitric oxide (NO) synthases resulting in the S-nitrosylation of GAPDH. This confers upon it the ability to bind to SIAH-1, and escort it to the nucleus where SIAH is then able to degrade key cellular proteins and initiate apoptosis. Taken together these observations suggest that SIAH-1 could play a similar role in breast carcinoma than in HHC cells depending on its expression level and sub-cellular localization.

Kid/KIF22 is a nuclear protein regulated by SIAH-1, whose level fluctuate in a cell cycle-dependent manner, increasing during pre-mitotic phases and greatly decreasing during mitosis [3]. Kid/KIF22 localized to the nucleus of the interphase cells upon entry into mitosis on condensed chromosomes and displayed some punctate cytoplasmic staining [21,34]. Interestingly, it was observed that SIAH-1 levels increased slightly during S-G2-M phases. SIAH-1 mediates Kid/KIF22 degradation via the ubiquitin-proteasome pathway and the balance between synthesis and degradation of these proteins influences the correct achievement of mitosis [3]. In the present study we observed a deregulation of both SIAH-1 and Kid/KIF22 proteins in tumor breast tissues, changing from a localized expression to a more diffuse pattern throughout the cell. Kid/KIF22 showed a different expression pattern in tumors compared to the normal tissue counterparts. Interestingly, in normal cells the protein was mostly localized in perinuclear areas whilst in malignant cells the expression was more diffuse and the punctuate staining pattern was mostly nuclear, possibly related to increased mitotic activity of these cells. In both the normal and tumor tissues we observed a similar cellular distribution pattern of both SIAH-1 and Kid/KIF22 staining consistent with previously described interaction and functional regulation between these two proteins.

The mRNA level of SIAHs and Kid/KIF22 showed an important variation among analyzed samples. In samples from the same patient, in most cases, SIAH-1 mRNA was down-regulated in tumoral breast tissues compared to surrounding normal breast tissues. Similar results about SIAH-1 expression have been reported in hepatocellular carcinomas [26,35], indicating that SIAH-1 mRNA expression is frequently reduced in malignant tissues compared to normal tissues. Matsuo *et al*. [26] observed that SIAH-1 was down-regulated in the majority of HCCs analyzed by semiquantitative RT-PCR, and SIAH-1 was not up-regulated in any of the cancerous tissues studied. It was also described using semiquantitative RT-PCR that SIAH-1 expression was lower in six hepatoma cell lines, compared to normal liver tissue [35]. Our study underlines the importance of relating the results of gene expression obtained by qRT-PCR to protein expression and the patterns of subcellular localization.

Given its structural similarity and possible redundant function with SIAH-1 we also analyzed the expression of SIAH-2 mRNA in our samples (data not shown). Although the median of mRNA copies of SIAH-2 was higher in normal than in tumour breast tissues, its expression was only decreased in half of tumour tissues compared to its normal counterpart. These different profiles suggest that pathways implicated in the control of the expression of these two members of the SIAH family could be different.

Kid/KIF22 mRNA expression showed also important differences among the samples. However, more interesting was the observed correlation between Kid/KIF22 mRNA variations between normal and tumor tissues when compared to SIAH-1 mRNA variations suggesting an additional regulation step at the level of gene transcription for these two interlinked proteins, in addition to the previously established mechanisms for protein stability. Although these data need to be confirmed using a larger number of samples, they suggest the existence of mechanisms of co-regulation for these two proteins that deserve to be explored.

## Competing interests

The authors declare that they have no competing interests.

## Authors' contributions

HBG and MM designed and coordinated the study and wrote the paper. HBG carry out biochemical and immunochemical studies. PF and LV carried out breast tissue collection and processing, and with M-PP and SM they participated in rtPCR studies. DP contributed with editing, and with MM and FC participated in the discussion and the writing of the paper

All authors read and approved the final manuscript.
